# *Allium sativum*, *Rosmarinus officinalis*, and *Salvia officinalis* Essential Oils: A Spiced Shield against Blowflies

**DOI:** 10.3390/insects11030143

**Published:** 2020-02-25

**Authors:** Stefano Bedini, Salvatore Guarino, Maria Cristina Echeverria, Guido Flamini, Roberta Ascrizzi, Augusto Loni, Barbara Conti

**Affiliations:** 1 Department of Agriculture, Food and Environment- University of Pisa, via del Borghetto 80, 56126 Pisa, Italy; stefano.bedini@unipi.it (S.B.); augusto.loni@unipi.it (A.L.); 2 Institute of Biosciences and Bioresources (IBBR), National Research Council of Italy (CNR), Corso Calatafimi 414, 90129 Palermo, Italy; salvatore.guarino@ibbr.cnr.it; 3 Facultad de Ingeniería en Ciencias Agropecuarias y Ambientales. Universidad Técnica del Norte, Av 17 de Julio 5-21, Ibarra 100105, Ecuador; mecheverria@utn.edu.ec; 4 Department of Pharmacy, University of Pisa, Via Bonanno 6, 56126 Pisa, Italy; guido.flamini@unipi.it (G.F.); roberta.ascrizzi@gmail.com (R.A.)

**Keywords:** blowflies, essential oil, repellent, insecticidal, bactericidal, fungicidal

## Abstract

Blowflies are known vectors of many foodborne pathogens and unintentional human ingestion of maggots by meat consumption may lead to intestinal myiasis. In fact, the control of insect pests is an important aspect of industrial and home-made food processing and blowflies (Diptera: Calliphoridae), which are among the most important pests involved in the damage of meat products. Most spices, largely used in food preparations and industry, contain essential oils that are toxic and repellent against insects and exert antimicrobial activity. In this study, we assessed the electro-antennographic responses, the oviposition deterrence, the toxicity, and the repellence of the essential oils (EOs) of *Allium sativum* L., *Salvia officinalis* L., and *Rosmarinus officinalis* L. against the blowfly *Calliphora vomitoria* L. We tested the EOs antibacterial and antifungal properties and the efficacy of an *A. sativum* EO-charged mist sprayed in the tunnel entryway of a meat processing room to form an olfactive barrier against the entrance of flies. The results showed that the EOs are perceived by female blowfly’ antennae and exert an evident repellent activity against them completely deterring the oviposition for up to 24 h starting from the concentration of 2.5 μL cm^−2^ EO. The EOs also exhibited toxic activity by both topical application (LD_50_ from 0.44 to 1.97 μL insect^−1^) and fumigation (LC_50_ from 1.76 to 31.52 μL L^−1^) against adults of *C. vomitoria* and were able to exert a clear antimicrobial activity toward pathogens. Lastly, the EO-charged mist was able to reduce by about 40% the presence of Calliphoridae in the meat processing room of a dry-ham factory.

## 1. Introduction

Blowflies (Diptera: Calliphoridae) are problematic synanthropic insect pests, which are important vectors of many pathogens. These flies, feeding on excrement and decaying organic matter, can easily transmit human and domestic animal pathogens to food and surfaces, causing the spread of foodborne illnesses and other diseases [1,2,3]. Blowflies are strongly attracted by the fresh meat on which they lay their eggs, which causes infestation by maggots and a rapid decaying due to the microorganisms they spread. In addition, in case of unintentional ingestion, blowfly maggots may also lead to human intestinal myiasis, an uncommon but severe pathology [4,5].

Besides fresh meat products, blowflies, especially the bluebottle fly *Calliphora vomitoria* L., represents a problem for the dry-cured meat and fish industry, as well. *C. vomitoria* infestation in the meat or fish industry is due to several peculiar characteristics of the species and, in particular, to its highly developed olfactory organs [6]. The infestation is often severe, due to the ability of females to lay hundreds of eggs in a very short time and to its very accelerated metabolism rate, which allows the fly to be active at low temperatures [7,8].

A traditional method to prevent pest infestation is the addition of plant-derived spices to the meat during its processing [9,10,11]. Spices have found wide applications both in the traditional food preparations and in the food industry for their flavouring, colouring, and, above all, preservative properties. Many compounds isolated from aromatic plants, such as essential oils (EOs), have shown antimicrobial activity against some of the most common microorganisms that affect the food quality and shelf-life as well as various beneficial effects for the consumers, including antioxidant and anti-inflammatory activities [12,13]. Even though EOs have also been proved to be effective as insecticides and repellents against food-stuff insect pests [14,15,16,17,18], very few information is available about their efficacy to protect fresh and processed meat from blowflies.

The aim of this work was to test the essential oils of *Allium sativum* L. (Amaryllidaceae), *Rosmarinus officinalis* L. (Lamiaceae), and *Salvia officinalis* L. (Lamiaceae), three aromatic plants commonly used in food processing, against the blowfly *C. vomitoria*. For this purpose, after determining their chemical composition, the EOs were first tested to determine the olfactory responsiveness of *C. vomitoria* by electro-antennography. Then we performed laboratory tests to determine their oviposition deterrence, ovicidal, and adulticidal effect. We tested the EOs for their antibacterial and antifungal properties against several common human pathogens. Lastly, we evaluated the repellence of a *A. sativum* EO-charged mist used as an olfactive barrier against the entrance of flies in a meat processing room of a dry-ham factory.

## 2. Materials and Methods 

### 2.1. Insects

Larvae of the bluebottle fly *C. vomitoria* were purchased from a commercial supplier (Fish Company Arco Sport, Cascina PI, Italy). The larvae were fed with beef liver and maintained at room temperature until pupation. The pupae were held in cages until the emergence of the adults, which were provided with a sugar-yeast diet (sugar and yeast 1:1) to provide the proteins amount necessary to stimulate oviposition [19,20] and water *ad libitum*. Species identification of the emerged adults was confirmed by an expert dipterologist (Alfio Raspi, former Professor, Department of Agriculture, Food and Environment, University of Pisa). *C. vomitoria* pupae and adults were kept under laboratory conditions (23 °C, 65 ± 5% R.H., natural photoperiod).

### 2.2. Essential Oils GC-MS Analysis

*A. sativum*, *R. officinalis*, and *S. officinalis* EOs were purchased from Vis Medicatrix Naturae s.r.l. (Florence, Italy). The EOs were chemically analysed by gas chromatography-electron impact mass spectroscopy (GC-EIMS) with a Varian CP-3800 gas chromatograph, equipped with a HP-5 capillary column (30 m × 0.25 mm, coating thickness 0.25 μm) and a Varian Saturn 2000 ion trap mass detector. The analytical conditions were as follows: injector and transfer line temperatures 220 °C and 240 °C respectively, oven temperature programmed from 60 °C to 240 °C at 3 °C/min, carrier gas helium at 1 mL/min, injection of 0.2 μL (10% hexane solution), and split ratio 1:30. Constituents identification was based on a comparison of retention times with those of authentic samples by comparing their LRIs with the series of *n*-hydrocarbons and using computer matching against commercial [21,22] and home-made library mass spectra (built up from pure substances and components of known oils and mass spectra literature data) [22,23]. 

### 2.3. Electroantennography (EAG)

The *A. sativum*, *R. officinalis*, and *S. officinalis* EOs were tested by electroantennogram recordings on *C. vomitoria* females (4–7 days old). The EOs were serially diluted 1:10 with ethyl alcohol (Sigma Aldrich, 99%) up to the concentration of 1 µg µL^−1^. One µL of each tested EO was used in aliquots ranging from 0.1 to 1000 µg (pure). The stimuli were pipetted on a piece of filter paper (Whatman No. 1), exposed to air for 5 min to allow the solvent to evaporate, and then inserted into a glass Pasteur pipette. *C. vomitoria* females used for this experiment were anesthetized with CO_2_ and their head was cut at the base. In order to avoid early saturation of the antennae, stimuli were tested from the lowest to the highest dose. For electroantennography (EAG) preparation glass capillary tubes filled with 0.1 M KCl solution were connected to the silver wire recording and reference electrodes. The recording electrode was placed gently by touching the tip of one randomly selected antenna, while the reference electrode was placed at the base of the head. A stimulus-flow controller (model CS-05, Synthech, the Netherlands) was used to generate a 3.0 s stimulus at a 1-min interval with a flow rate of 1.5 L min^−1^. The antennal responses were passed through a high impedance amplifier (model IDAC-4, Synthech, Hilversum, the Netherlands) and recorded with specialized software (Synthech). At the beginning and the end of the stimulation of the antenna with each plant essential oil, 1 µL of pure ethyl alcohol was puffed as a control stimulus. The same antenna was used to test all of the EOs at all the concentrations. Each EO was tested on 10 female antennae using one antenna per fly (N = 10). The sequence of the tested essential oil was randomized.

### 2.4. Oviposition Deterrence

The oviposition deterrence of the essential oils against *C. vomitoria* was evaluated by exposing fresh meat treated with the EOs to *C. vomitoria* mature females in a cage. In detail, 150 *C. vomitoria* unsexed adults 10 to 14-days-old were placed into a cage (47.5 L × 47.5 W × 93 cm H) (model BugDorm-44590 Insect Rearing Tent) with net sides and front mesh ends. The cage was collocated under a bank of fluorescent lamps to provide even lighting and were maintained at about 23 °C and 65 ± 5% R.H. A beaker containing 500 mL of water, covered by a net, was placed in the cage to provide humidification. Oviposition stimulus was provided by polyethylene embedding moulds (Peel-A-Way^®^ Embedding Mold (Truncated - T12) (Polysciences Europe GmbH, Hirschberg an der Bergstrasse, Germany) filled by pork meat (5 g) mixed with 1 mL of water to prevent desiccation. The meat surface (4.84 cm^−2^) was gently pressed and treated by a glass nebulizer with 100 μL of ethanolic EO solution at 0% (control), 0.5%, 1%, and 2%, corresponding to 0, 1.2, 2.5, and 5 μL cm^−2^ of meat. At each corner of the cage, four meat-moulds treated with the four different EO concentrations (0, 1.2, 2.5, and 5 μL cm^−2^) were placed on a Petri dish lid about 3 cm from the edge of the cage. To minimize any border effect, the reciprocal disposition of the EO concentrations was maintained at the four cage corners. A scheme of the disposition of the meat-moulds inside of the cage is reported in Figure S1.

To evaluate the intensity and the lasting effect of the Eos’ protection, the eggs laid onto the meat were counted after 3, 24, 48, and 72 h. Eggs were counted under a dissection microscope. The number of eggs laid in large aggregates was counted by an analytical balance equipped with a piece counter function. The number of eggs laid onto the four meat moulds treated with the same EO concentration were summed together. The experiment was performed with three replicates (one cage for replicate).

Oviposition deterrence was evaluated by calculating the Oviposition Activity Index (OAI) using the formula below.

OAI = (NT − NC)/(NT + NC)

where NT = total number of eggs on the treated meat and NC = total number of eggs on the control meat [24]. For OAI values ≤ -0.3, the EO was considered as repellent [25].

### 2.5. Ovicidal Bioassay

The toxicity of the essential oils against the *C. vomitoria* eggs was evaluated by exposing freshly laid eggs to several concentrations of the three EOs. One-hundred eggs were placed in a petri dish on filter paper (4 cm Ø) treated with 100 µL of 0.25%, 0.50%, 0.75%, 1.00%, 2.00%, 5.00%, and 10.00% EtOH solutions of the EOs corresponding to 0.02, 0.04, 0.06, 0.08, 0.16, 0.40, and 0.80 µL cm^−2^. As a form of control treatment, 100 eggs were placed on filter paper treated with 100 µL of EtOH only. For all the treatments, before placing the eggs, ethanol was evaporated by exposing the treated paper to an airflow for 3–5 min. For each concentration, three replicates, with each one of 100 eggs, were performed.

### 2.6. Adulticidal Bioassays

The toxicity of the essential oils against the *C. vomitoria* adults was evaluated both by fumigation and topical application bioassays. For the fumigation bioassay, 10 unsexed adult flies were placed in an airtight glass jar (300 mL) with a screw lid. A piece of filter paper was adhered inside the lid. Furthermore, 100 µL of 0.25%, 0.5%, 1%, 5%, and 10% EtOH solutions of the EOs, corresponding to 1, 2, 3, 17, and 33 µL L^−1^ of air, were applied to the filter paper. The treated filter paper was protected from direct contact with the insect by a thin layer of sterile gauze. The control jars were treated with 100 µL of EtOH only. For all the treatments, before placing the lid, ethanol was evaporated by exposing the treated paper to an airflow for 3–5 min. The jars were further sealed with Parafilm and maintained under laboratory conditions (23 °C, 65 ± 5% R.H.). Each test was replicated three times and mortality was checked at 24 h. As for the toxicity tests by topical application, the three EOs were tested against 7–10 days-old adults of *C. vomitoria*. Flies were treated by a Burkard hand micro-applicator equipped with a one-mL syringe. Two µL of 10%, 20%, and 40% EtOH solutions of the EOs (corresponding to 0.2, 0.4, and 0.8 µL insect^−1^) were applied on the thorax of 10 unsexed adult flies [26]. Three replicates (30 treated flies) were run for each dose. Control flies were treated with 2 µL of EtOH only. Insects were maintained in Plexiglas cages of 20 cm of diameter and 30 cm long (10 insects per cage) with water and sugar *ad libitum* under laboratory conditions. Mortality of the flies was checked after 24 h and values were corrected using the Abbott formula [27].

### 2.7. Antimicrobial Assays

The EOs were individually tested against *Escherichia coli* ATCC 10536, *Staphylococcus aureus* (ATCC BAA-1026), *Bacillus subtilis* (ATCC 11774), *Salmonella enterica* subsp. *enterica* serovar Abaetetuba (ATCC 35640), *Pseudomonas aeruginosa* (ATCC 15442), and *Candida albicans* (ATCC 10231). All the strains were purchased from the American Type of Culture Collection (ATCC, Manassan, USA) and maintained in the Laboratories of the Universidad Técnica del Norte, Ecuador. *E. coli*, *S. aureus*, *P. aeruginosa*, and *B. subtilis* strains were grown on nutrient agar. *C. albicans* strain was grown on malt agar. *S. enterica* was grown on trypticase soy agar.

The antibacterial activity of the EOs was determined by assessing the minimum inhibitory concentration (MIC) and minimum lethal concentration (MLC) following the method described by Bedini et al. [28]. In brief, 5 mL of 10^7^ UFC mL^−1^ microbial broth was incubated in tubes containing 50 μL of decreasing concentrations of the EO (10, 5, 2.5, 1.25, and 0.63 μL per tube). The MIC was estimated as the lowest EOs concentration that inhibited any visible microbial growth [29]. The MLC was calculated as the highest EO dilution at which no growth occurred on the plates. Microorganisms’ death was checked by sub-culturing 0.1 mL of the cell suspensions from the tubes by showing no growth on nutrient agar plates for bacteria and on malt agar plates for yeast. Three repetitions of each treatment were made.

### 2.8. EO-Charged Mist Assay

The *A. sativum* EO, which resulted as the most effective EOs in the oviposition deterrence, was tested as an active ingredient in a repellent mist sprayed by an automatic system. The repellent activity of the EO-charged mist was tested in a ham factory (Micheletti S. r. l., Lammari, Lucca, Italy). A stable water emulsion of the *A. sativum* EO was obtained by mixing the EO with 1% Tween 80 water solution to a final EO concentration of 1%. The emulsion was automatically mixed with tap water and sprayed by an automatic system (Gardensystem, Freezanz System Srls, Pisa, Italy) through nine 0.3-mm brass misting nozzles located in order to form an olfactory barrier (EO-charged mist) at the entrance tunnel (5 L × 2.6 W × 4 H m) of the meat processing room. The spraying system was active from 6 a.m. to 18 p.m. for 5 s every 10 min. The quantity of the emulsion sprayed was 100 mL day^−1^. The effectiveness of the mist as a barrier against the entrance of the flies was assessed by a fly light trap (Mantis 1 × 2, PestWest U.S.A. LLC., Sarasota, USA) equipped with a glue board (419 × 23 cm) (Boacha 850, PestWest U.S.A. LLC., Sarasota, USA) that was placed inside the meat processing room. The glue board of the trap was removed weekly and transported in the lab for identifying the trapped Diptera. A 0.1% Tween 80 water solution spray (Control mist) was used as control treatment. The EO-charged mist or the Control mist treatments were performed every other week for 6 weeks to obtain three replicates for each treatment. The identification of captured specimens belonging to Muscidae and Calliphoridae was performed by adopting the key of Oosterbroek [30] for European families of Diptera.

### 2.9. Statistics and Data Analyses

Log-probit regressions were used to assess the toxicity of the EOs against *C. vomitoria* adults. LD_50_ and the LC_50_ values of the two EOs were reported as toxicological endpoints for comparison. Significant differences between the toxicity regressions were determined by estimating confidence intervals of the relative median potency (rmp). Differences between toxicity values were considered statistically significant when values in the 95% confidence interval of relative median potency analyses were ≠ 1.0. Oviposition deterrence and toxicity percentage data were transformed into arcsine values before statistical analysis. Oviposition deterrence data were processed by one–way between–groups univariate analysis of covariance (ANCOVA) with the EO as a fixed factor. The EOs concentration and the time of exposition were considered as covariates in the model and their effect was controlled in the analysis. Post hoc comparisons were performed using Bonferroni corrections for multiple comparisons. The estimated marginal (EM) means of the insect pests’ mortality are reported. Electro-antennographic data were analysed by performing a two-way ANOVA with the EO and the concentration as fixed factors after normalizing the data for the control. Differences between solvent and the EOs concentrations among each EO were assessed by one-way ANOVA, which was followed by a Fisher’s least significant difference (LSD) test. The differences in the sizes of the inhibitory zones formed by the EOs against different microbial strains were tested by the Kruskal-Wallis test and the means were separated by Dunn-Bonferroni pairwise comparisons. The independent-samples t-test (two-tailed) was used to assess differences between the mean number of flies trapped during the treatment with the essential oil mist (EO-charged mist) and tween-80 only (Control) during the EO-charged mist assay. The ANOVA and ANCOVA assumptions of variance homogeneity and normality were confirmed by Levene’s test and the Shapiro-Wilk test, respectively. Statistics were performed by SPSS 22.0 software (IBM SPSS Statistics, Armonk, North Castle, NY, USA).

## 3. Results

### 3.1. Chemical Composition of the EOs

The complete compositions of the three EOs are reported in Table 1. Overall, 99 compounds were identified. The EOs of *R. officinalis* and *S. officinalis*, both belonging to the Lamiaceae family, showed compositional similarities while *A. sativum* EO exhibited a completely different profile.

Sulfur compounds dominated (over 90%) the *A. sativum* EO composition: diallyl trisulfide was the most abundant one, which was followed by diallyl tetrasulfide and diallyl disulfide.

The two Lamiaceae EOs, instead, were rich in oxygenated monoterpenes for which similar relative abundances were detected: 1,8-cineole and α-thujone were the most represented compounds in *R. officinalis* and *S. officinalis* EOs, respectively. Other compounds of this chemical class showing relevant presence in both the compositions were camphor and borneol. Monoterpene hydrocarbons are followed for both these species’ EOs as the second most abundant chemical class of compounds: for both α-pinene and β-pinene, and camphene were detected in relevant relative abundances (over 2.5%).

### 3.2. Electroantennography (EAG)

**Figure 1:**
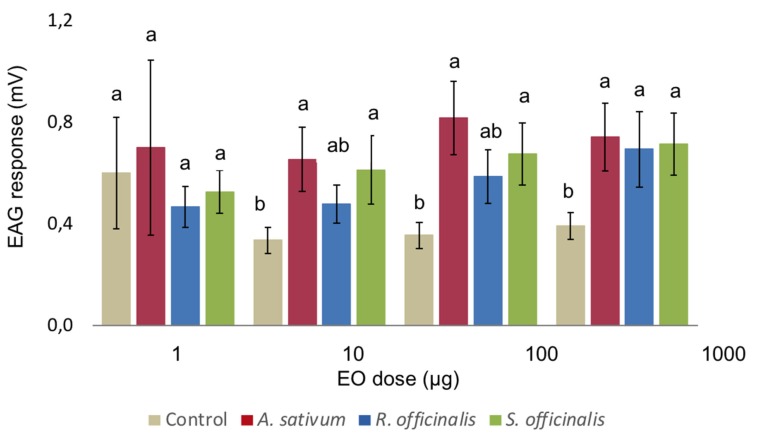
Mean Electroantennography responses of female *C. vomitoria* to puff test using 1 µL of ethanol solution containing different concentrations of *Allium sativum*, *Rosmarinus officinalis,* and *Salvia officinalis* essential oils. Bars indicate standard error. Values labelled with different letters are significantly different by an LSD test (*p* ≤ 0.05).

The results of Electroantennography (EAG) bioassays are reported in Figure 1. Starting from 10 µg µL^−1^, *A. sativum* and *S. officinalis* EOs elicited a significantly higher EAG response on the antennae of *C. vomitoria* female when compared to the solvent. On the contrary, *R. officinalis* EO elicited a significant response only at 1000 µg µL^−1^ (Figure 1). Overall, the two-way ANOVA showed a significant effect of the EO (*F*_2,72_ = 5.646, *p* = 0.005), while no significant difference where determined by the concentration (*F*_3,72_ = 1.952, *p* = 0.129) with no interaction of the two factors (*F*_6,72_ = 0.232, *p* = 0.965).

### 3.3. Oviposition Deterrence

**Figure 2:**
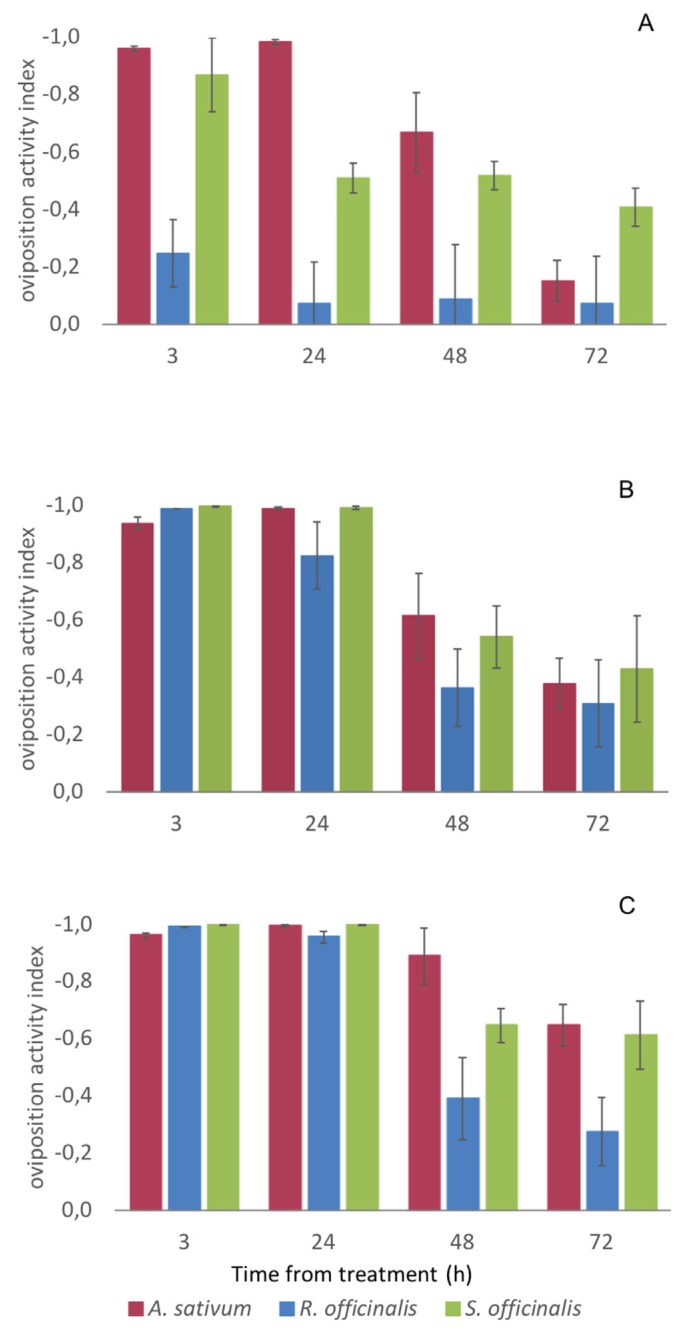
Oviposition deterrence of *Allium sativum*, *Rosmarinus officinalis,* and *Salvia officinalis* essential oils (EOs) against *Calliphora vomitoria* females. (**A**), 1.2, (**B**) 2.5, and (**C**), 5 μL EO cm^−2^ of meat. For values ≤ - 0.3, the EO is considered significantly repellent [25]. Bars represent a standard error.

At the highest concentration (5 μL cm^−2^ EO), all the three tested EOs showed a significant oviposition deterrence up to 72 h (OAI ≤ −0.3) and they were all able to completely avoid the oviposition on the meat by *C. vomitoria* up to 24 h. A complete suppression of the oviposition was also observed in the meat treated with 2.50 μL cm^−2^ of *A. sativum* and *S. officinalis* EO whereas, at 1.25 μL EO cm^−2^, only the EO of *A. sativum* was able to completely deter *C. vomitoria* oviposition up to 24 h (Figure 2).

Overall, the ANCOVA showed a significant difference among the oviposition deterrence of the three EOs after controlling for the effects of the EO concentration and time of exposure (*F*_2, 139_ = 10.688, *p* < 0.001, *η_p_^2^* = 0.133). In detail, Bonferroni post-hoc tests of the OAI EM means indicated no significant differences between *A. sativum* and *S. officinalis* EOs (OAI EM means = −0.574 ± 0.037, and −0.534 ± 0.037, respectively, *p* = 1.000) and that the two were significantly more effective than the *R. officinalis* one (OAI EM mean = −0.349 ± 0.037, *p* = 0.002) (Table 2).

### 3.4. Ovicidal Bioassays

**Figure 3:**
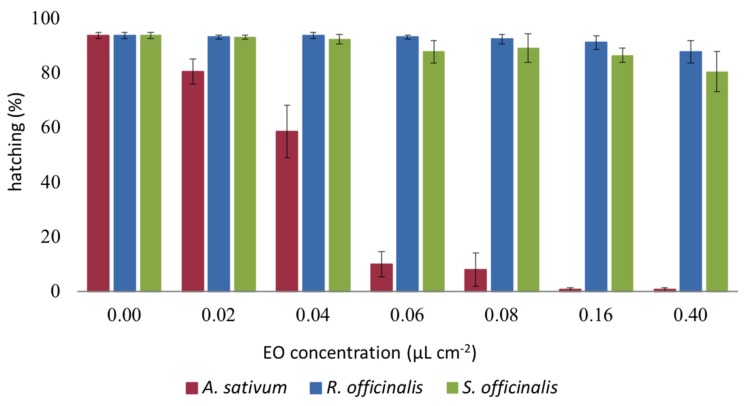
Ovicidal effect of *Allium sativum*, *Rosmarinus officinalis,* and *Salvia officinalis* essential oils. Data are expressed as a mean percentage of eggs hatching. Bars represent the standard error.

After 24 h, 94% of control eggs hatched while we observed an inhibition of hatching of the eggs treated with the EOs with differences depending on the EOs (*F*_2,48_ = 452.72, *p* < 0.001), the concentration (*F*_7,48_ = 45.268, *p* < 0.001), and interaction EOs *x* concentration (*F*_14,48_ = 21.894, *p* < 0.001). Among the three tested EOs, the *A. sativum* one was the most effective (Tukey’s hsd test, *p* < 0.001) with a complete inhibition of the hatching starting from 0.16 μL EO cm^−2^ (Figure 3).

### 3.5. Adulticidal Bioassays

**Figure 4:**
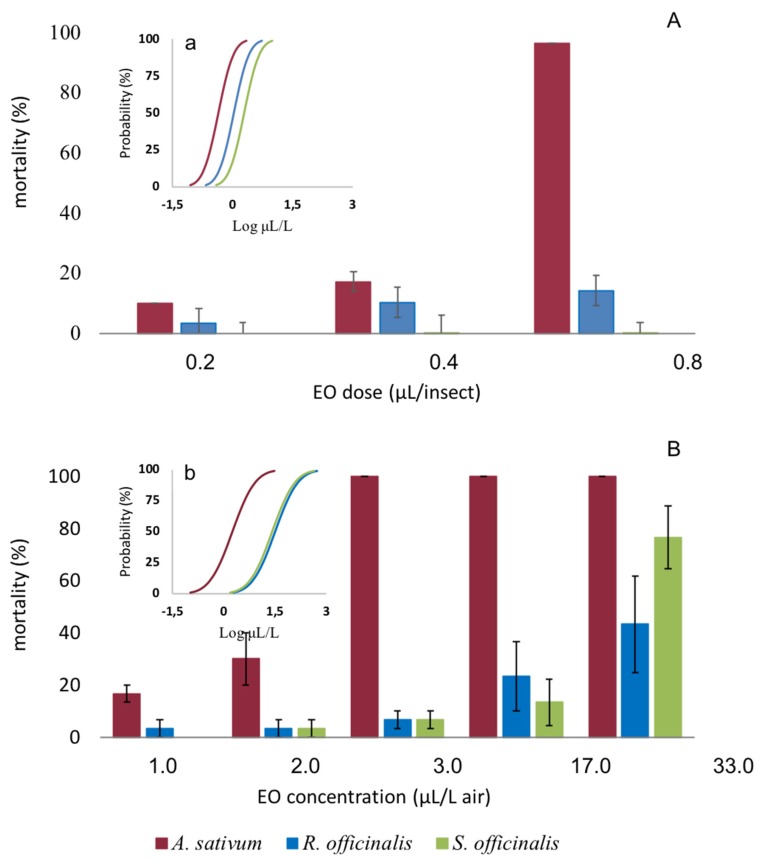
Mean mortality of *Calliphora vomitoria* adults exposed by topical application (**A**) and fumigation (**B**) to *Allium sativum*, *Rosmarinus officinalis,* and *Salvia officinalis* essential oils. Bars represent the standard error. Embedded graphics show the probability of mortality of the insects exposed to the EOs by topical application (a) and by fumigation (b) calculated by Log-Probit regression.

The EOs exerted toxic activity by both topical application and fumigation against adults of *C. vomitoria* (Figure 4).

By using a topical application, the toxicity depended on the EOs (*F*_2,18_ = 48.560; *p* < 0.001), the dose (*F*_2,18_ = 38.743, *p* < 0.001), and, with significant interaction, EOs *x* dose (*F*_4,18_ = 13.530; *p* < 0.001). Post-hoc tests indicated a significant higher toxicity of *A. sativum* compared to *R. officinalis* and *S. officinalis* EOs (Tukey’s hsd test, *p* < 0.001) and a higher toxicity of *R. officinalis* than *S. officinalis* EOs (Tukey’s hsd test, *p* < 0.017). Median lethal dose (LD_50_) values calculated by Probit analysis were 0.44, 1.10, and 1.97 μL L^−1^ air for *A. sativum*, *R. officinalis*, and *S. officinalis* EOs, respectively (Table 3). RMP analysis showed that *A. sativum* was significantly more effective than the *R. officinalis* (*A. sativum* vs. *R. officinalis* RMP = 0.400, 95% CI: 0.007, 0.922) and the *S. officinalis* EOs (*A. sativum* vs. *S. officinalis* RMP = 0.224, 95% CI: 0.000, 0.738).

By fumigation, the *A. sativum* EO killed all the fly population starting from 3.0 μL L^−1^ air (Figure 4). The toxicity of the EOs was dependent on the EOs (*F*_2,36_ = 65.117, *p* < 0.001), the concentration (*F*_5,36_ = 36.938, *p* < 0.001), and with significant interaction EOs *x* concentration (*F*_10,36_ = 7.299, *p* < 0.001). We observed a much higher activity of the *A. sativum* EO with respect to the others (Tukey’s hsd test, *p* < 0.001). Median lethal concentration (LC_50_) values calculated by Probit analysis were 1.76, 31.52, and 25.52 μL L^−1^ air for *A. sativum*, *R. officinalis*, and *S. officinalis* EOs, respectively (Table 4). RMP analysis showed that *A. sativum* was significantly more effective than the *R. officinalis* (*A. sativum* vs. *R. officinalis* RMP = 0.056, 95% CI: 0.00, 0.367) and *S. officinalis* EOs (*A. sativum vs S. officinalis* RMP = 0.069, 95% CI: 0.00, 0.421).

An overall significant linear relationship between the concentration/dose of the EOs and the mortality of the flies was observed by both fumigation and topical application (*R* = 0.636, *F*_1, 52_ = 35.254, *p* < 0.001, and *R* = 0.522, *F*_1, 52_ = 9.374, *p* = 0.005 for fumigation and topical application, respectively).

### 3.6. Antimicrobial Activity

The MIC and MLC values showed that the most generally susceptible microbial pathogens were *B. subtilis* and *S. aureus* with MIC and MLC values for *A. sativum* and *S. officinalis* EOs lower than 0.63 μL mL^−1^. The most resistant microbial strain was *S. enterica* with values of 10 μL mL^−1^ or above for all the three EOs (Table 5).

### 3.7. EO-Charged Mist Assay

**Figure 5:**
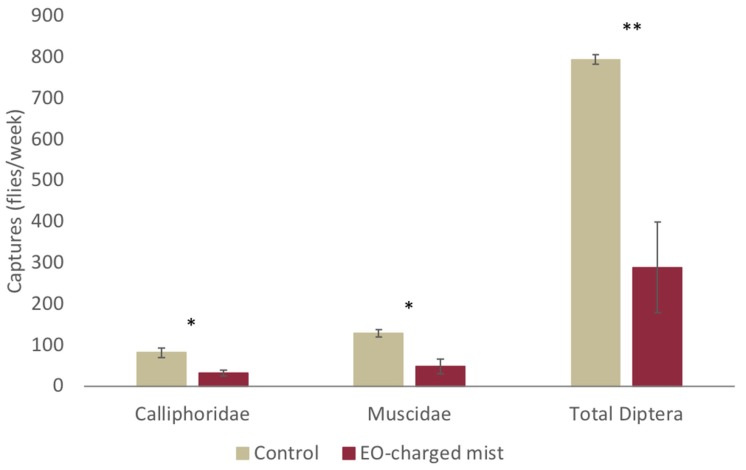
Mean number of flies trapped inside the meat processing room during the treatment of the plant entrance tunnel by the EOs-charged or control mist. Bars represent a standard error. Asterisks indicate a significant difference between the treatments for each category by t-test (*, *P* ≤ 0.05; **, *P* ≤ 0.01).

The olfactive barrier created by the EO-charged mist significantly reduced the number of Diptera captured inside of the meat processing plant (Figure 5). The t-test showed that the Calliphoridae (t_4_ = 3.590, *p* = 0.023) and the Muscidae (t_4_ = 4.061, *p* = 0.015) as well as the Total Diptera trapped (t_4_ = 4.591; *p* = 0.010) were significantly lower when the EO emulsion was sprayed at the entrance of the processing plants.

## 4. Discussion

EO and spice extracts have found widespread use in the food industry not only for their aroma and colouring, but also for their preservative properties and for the beneficial effects for the health of consumers [13,31]. However, despite the fact that it has been shown that spice EOs are also toxic and repellent against food insect pests, very little is known today about their use against synanthropic flies. In this case, for the first time, we tested the EOs of three of the most widely used spices as insecticides and repellents against the blowfly *C. vomitoria* a pest of meat products and a vector of foodborne diseases.

The chemical compositions of the EOs tested in this study are consistent with the results obtained in the EAG assay. Generally, since the EAG response is linked to the insect olfaction, it is reasonable that this was primarily elicited by the more volatile fraction of the extracts as reported for other dipteran species [32,33]. In our experiments, the *A. sativum* EO elicited an overall stronger EAG response than the other two EOs tested. Nevertheless, there was not a clear concentration dependent response, as observed in other dose response experiments, using EOs as stimuli [34]. Compared with the solvent, *A. sativum* and *S. officinalis* EOs determined significant antennal responses from the concentration of 10 µg µL^−1^, while EAG responses at 1 µg µL^−1^ were similar to the solvent, which indicates a threshold of perception for these EOs concentrations. In the case of the *A. sativum* EO, such a prompt response elicited on the *C. vomitoria* female antennae could be determined by the relatively high amount of sulphur compound volatiles (about 94%) in the EO composition. In fact, *Calliphora* spp. flies are known to be very sensitive to sulphur compounds as dimethyl trisulphide [35,36]. On the other hand, the EAG responses elicited on *C. vomitoria* antennae by the *S. officinalis* EO are more likely determined by the terpenes fraction as reported for the housefly *Musca domestica* L. [37].

The responsiveness of *C. vomitoria* to the three EOs was confirmed by the results of the oviposition deterrence bioassay that showed that *A. sativum*, *R. officinalis*, and *S. officinalis* EOs are able to exert a clear deterrent activity against *C. vomitoria* females’ oviposition. Such results are in good agreement with previous experiments showing a strong oviposition deterrence by *Artemisia dracunculus* L. and *A. annua* L. (Asteraceae) EOs [19] against *C. vomitoria* and of *Lavandula angustifolia* Mill., and *Clinopodium nubigenum* (Kunth) EOs against *Lucilia sericata* Meigen (Diptera Calliphoridae) [28]. A repellent effect of EOs was also observed by Callander and James [38] who obtained the complete inhibition of oviposition of *L. cuprina* on wool treated with tea tree (*Melaleuca alternifolia* Maiden & Betche) EO. In comparison with the *Artemisia* species, the EOs extracted from the three spices utilized in this work were less effective as oviposition deterrent. However, it should be highlighted that the use of EOs extracted from aromatic plants widely used as spices has the advantage that they are allowed in the food industry as ingredients [39]. In addition to oviposition deterrence, we also observed a strong toxicity of the *A. sativum* EO against adults and eggs of *C. vomitoria* that was significantly higher than those of the *R. officinalis* and *S. officinalis* ones. In line with our results, the *A. sativum* extract was recently found to be toxic to blowflies’ larvae [40] and a clear ovicidal action of *A. sativum* EO has also been reported on the coleopteran *Callosobruchus maculatus* F. [41]. The higher bioactivity against *C. vomitoria* of the *A. sativum* among the tested EOs could be due to the presence of diallyl trisulfide and methyl allyl trisulfide, which are the *A. sativum* EO main compounds. Such substances were isolated and tested for their bioactivities as oviposition deterrent and adulticidal agents against the coleopteran *Sitophilus zeamais* Motschulsky and *Tribolium castaneum* Herbst. Both the compounds inhibited or reduced the egg hatching. Moreover, diallyl trisulfide and methyl allyl trisulfide showed adulticidal activity both by contact and fumigation as well as anti-feedant activity [42]. In the present study, however, a holistic approach without isolation of the main compounds has been preferred and the entire EO has been used. Published literature reports higher efficacy for complete EOs, including their minor compounds, whose presence seems synergic for the overall bioactivity [43,44]. On the other hand, as already mentioned above, the use of the whole EO instead of its purified active compounds may be preferable since EOs are natural extracts of spices allowed in the food production.

The antimicrobial assays performed in this study showed that the EOs besides their toxic and repellent effect against the insect pests are also able to exert a strong antimicrobial activity against various bacterial and fungal human pathogens. In particular, among the foodborne disease bacteria, we observed a strong activity against *E. coli*, while *S. enterica* was the most resistant strain. In line with these results, *S. enterica* was found to be the most resistant microbial strain for the EOs extracted from *C. nubigenum* and *L. angustifolia* [28]. Such antimicrobial activity of the EOs is important since Calliphoridae are vectors of numerous microorganisms and foodborne pathogens [1,2,3]. Landing on the meat for feeding or for oviposition, they can easily contaminate the food, which causes the spread of foodborne diseases and the rotting of the product.

On the basis of the results obtained in the oviposition deterrence and toxicity bioassays, we have selected the *A. sativum* EO to be tested as an active ingredient in an EO-charged mist sprayed in the entryway tunnel of a ham factory in order to form an olfactory barrier able to control the entrance of the flies in the meat processing room. This usually occurs when the doors are open to allow the unloading of the fresh meat and the loading of the hams. Even if EOs have been extensively studied as topical repellent against insects of medical or veterinary importance [20,45,46,47], and proposed as repellents in food packaging [48,49,50,51] and stored foodstuff [18,44,52], to the best of our knowledge, this is the first report on the use of an EO-charged mist as an olfactory barrier to protect an indoor working space from the entrance of problematic flies. In line with our results, however, a good efficacy of the EOs as aerosol spray have been reported by Revay et al. [53] who observed that a timed-released 0.3% aqueous geraniol emulsion significantly reduced mosquito-biting pressure of *Culex pipiens* L. and *Aedes albopictus* Skuse (Diptera: Culicidae) in outdoor conditions. Compared to a direct use of the EOs as treatment of the food, the EO-charged mist has the advantage that the EO is not in direct contact with the food and, by consequence, there is no alteration of its chemical and organoleptic properties. 

## 5. Conclusions

Insect pests and food-borne diseases represent a growing economic and public health problem worldwide. Thanks to their high availability, low cost, and acceptance as ingredients in food preparation, spice EOs may represent excellent repellents and insecticides to be used in food processing plants. Our results indicate that selected spice EOs may act as an effective low-cost natural shield able to control the presence of problematic flies into food processing rooms in order to protect meat and fish products from blowflies’ infestation and microbial contamination by both direct application on food and sprayed to form an olfactory barrier.

## Figures and Tables

**Table 1:** Chemical composition of *Allium sativum*, *Rosmarinus officinalis,* and *Salvia officinalis* essential oils.

Compounds	l.r.i.^a^	Relative Abundance (%)		
		*A. sativum*	*R. officinalis*	*S. officinalis*
(*Z*)-3-hexen-1-ol	855	- ^b^	Tr ^c^	-
(*Z*)-salvene	855	-	-	tr
(*E*)-2-hexenal	856	-	tr	-
diallyl sulfide	866	5.5	-	-
(*E*)-salvene	866	-	-	tr
2,3-dimethyl thiophene	901	0.3	-	-
santolina triene	909	-	tr	-
methyl-2-propenyl disulfide	920	3.6	-	-
tricyclene	928	-	0.2	0.7
α-thujene	931	-	0.3	0.7
(*Z*)-methylpropenyl disulfide	932	0.2	-	-
(*E*)-methylpropenyl disulfide	940	0.2	-	-
α-pinene	941	-	9.8	4.6
camphene	954	-	4.2	5.7
thuja-2,4(10)-diene	959	-	tr	-
dimethyl trisulfide	975	0.8	-	-
sabinene	976	-	tr	tr
1-octen-3-ol	980	-	tr	-
β-pinene	982	-	5.6	2.5
3-octanone	987	-	tr	-
myrcene	993	-	1.5	1.1
α-phellandrene	1005	-	0.3	0.2
δ-3-carene	1011	-	tr	-
α-terpinene	1018	-	0.8	tr
*p*-cymene	1027	-	1.8	1.1
limonene	1032	-	-	tr
1,8-cineole	1034	-	41.2	11.9
(*Z*)-β-ocimene	1042	-	tr	-
(*E*)-β-ocimene	1052	-	tr	-
γ-terpinene	1062	-	0.9	1.8
*cis*-sabinene hydrate	1070	-	tr	tr
*trans*-linalool oxide (furanoid)	1076	-	tr	tr
camphenilone	1078	-	tr	-
diallyl disulfide	1082	16.1	-	-
terpinolene	1088	-	0.5	0.9
*p*-cymenene	1089	-	tr	-
linalool	1101	-	1.4	-
*(E)*-1-allyl-2-(prop-1-en-1-yl) disulfane	1103	0.7	-	-
α-thujone	1104	-	-	22.2
*(Z)*-1-allyl-2-(prop-1-en-1-yl) disulfane	1107	0.6	-	-
β-thujone	1118	-	-	5.5
*exo*-fenchol	1119	-	tr	-
α-campholenal	1125	-	tr	-
*trans*-pinocarveol	1139	-	tr	-
nopinone	1140	-	tr	-
methyl allyl trisulfide	1142	9.5	-	-
camphor	1143	-	11.7	16.2
4-methyl-1,2,3-trithiolane	1154	0.9	-	-
*trans*-pinocamphone	1162	-	tr	-
pinocarvone	1163	-	tr	-
borneol	1165	-	4.7	4.2
*cis*-pinocamphone	1175	-	tr	-
4-terpineol	1178	-	1.1	0.6
*p*-cymen-8-ol	1183	-	tr	-
α-terpineol	1189	-	3.3	0.1
myrtenol	1193	-	-	tr
decanal	1204	-	tr	-
verbenone	1205	-	tr	-
2-vinyl-4H-1,3-dithiine	1206	0.6	-	-
dimethyl tetrasulfide	1210	0.8	-	-
*endo*-fenchyl acetate	1223	-	-	tr
isobornyl formate	1232	-	tr	-
3-methyl-3-hexen-1-yl butanoate	1236	-	-	tr
carvotanacetone	1248	-	-	tr
*iso*bornyl acetate	1285	-	1.5	2.8
*trans*-sabinyl acetate	1291	-	-	tr
diallyl trisulfide	1297	23.1	-	-
(*Z*)-1-allyl-3-(prop-1-en-1-yl) trisulfane	1329	0.2	-	-
(*E*)-1-allyl-3-(prop-1-en-1-yl) trisulfane	1346	0.6	-	-
α-cubebene	1351	-	-	tr
S-methyl-1,2,3,4-tetrathiane	1364	1.0	-	-
α-ylangene	1372	-	tr	-
α-copaene	1376	-	tr	0.2
S-propylpropane thiosulfonate	1388	6.7	-	-
methyl eugenol	1403	-	tr	-
(*Z*)-caryophyllene	1405	-	tr	tr
β-caryophyllene	1420	-	7.4	7.9
1-(1-(methylthio) propyl)-2-propyl disulfane	1431	0.5	-	-
aromadendrene	1445	-	-	tr
dimethyl pentasulfide	1450	0.3	-	-
α-humulene	1456	-	1.2	8.3
(*E*)-β-farnesene	1460	-	tr	-
*allo*aromadendrene	1461	-	-	tr
*trans*-cadina-1(6),4-diene	1470	-	-	tr
γ-muurolene	1477	-	tr	-
viridiflorene	1496	-	-	tr
β-bisabolene	1509	-	0.3	-
*trans*-γ-cadinene	1513	-	tr	-
δ-cadinene	1524	-	0.2	-
diallyl tetrasulfide	1540	17.4	-	-
caryophyllene oxide	1581	-	0.3	0.2
1-propyl-2-(4-thiohept-2-en-5-yl) disulfide	1581	0.2	-	-
viridiflorol	1590	-	-	0.4
6-methyl-4,5,8-trithia-1,10-undecadiene	1597	0.9	-	-
humulene epoxide II	1607	-	tr	0.2
3-amino-tert-butyl benzoate	1620	0.9	-	-
1-allyl-3-(2-(allylthio)propyl) trisulfane	1818	2.0	-	-
cyclic octaatomic sulfur	2030	0.2	-	-
1-allyl-3-(2-(allyldisulfanyl)propyl) trisulfane	2066	1.2	-	-
Monoterpene hydrocarbons		-	25.9	19.3
Oxygenated monoterpenes		-	64.9	63.5
Sesquiterpene hydrocarbons		-	8.9	16.4
Oxygenated sesquiterpenes		-	0.3	0.8
Nitrogen compounds		0.9	-	-
Phenylpropanoids		-	tr	-
Sulfur compounds		94.1	-	-
Other non-terpene derivatives		-	tr	tr
Total identified (%):		95.0	100.0	100.0

^a^, Linear retention indices on a DB5 column, ^b^, Not detected, ^c^, Traces, < 0.1%.

**Table 2:** Adjusted estimated marginal (EM) means of the oviposition activity index (OAI) of *Calliphora vomitoria* females on meat treated with *Allium sativum*, *Rosmarinus officinalis,* and *Salvia officinalis* essential oils (EOs).

EOs	Mean^a^ ± SE	95% Confidence Interval	
		Lower Bound	Upper Bound
*A. sativum*	−0.574 ± 0.037	−0.646	−0.501
*R. officinalis*	−0.349 ± 0.037	−0.422	−0.277
*S. officinalis*	−0.534 ± 0.037	−0.606	−0.461

^a^ Data are expressed as OAI ± standard error. Covariates (dose and time of exposure) are evaluated at the following values: EOs concentration = 0.875 μL EO cm^−2^ of meat, Time = 36.75 h.

**Table 3:** Toxicity by topical application of *Allium sativum, Rosmarinus officinalis,* and *Salvia officinalis* essential oils (EOs) against adults of *Calliphora vomitoria*.

EO	LD_50_^a^	LD_95_^b^	Intercept	*p*
*A. sativum*	0.44(0.23–1.02)	1.37(0.72–56.79)	1.186	< 0.001
*R. officinalis*	1.10(0.60–10.70)	3.43(1.40–807.14)	−0.138	0.544
*S. officinalis*	1.97(0.79–74.88)	6.13(1.87–5502.96)	−0.978	0.001

^a^, Concentration of the EO that kills 50% of the exposed flies. ^b^, Dose of the EO that kills 95% of the exposed flies. Data are expressed as µL insect^−1^. In bracket, confidence interval. Pearson goodness of fit test: *χ2* = 16.353, df = 5, *p* = 0.006. Since the significance level (*p*) is less than 0.150, a heterogeneity factor is used in the calculation of confidence limits.

**Table 4:** Toxicity by fumigation of Allium sativum, Rosmarinus officinalis, and Salvia officinalis essential oils (EOs) against adults of Calliphora vomitoria.

EO	LC_50_^a^	LC_95_^b^	Intercept	*p*
*A. sativum*	1.76(0.62–4.12)	13.04(5.30–109.21)	−0.467	0.002
*R. officinalis*	31.52(12.87–133.39)	232.90(70.04–5513.08)	−2.838	<0.001
*S. officinalis*	25.52(10.39–96.36)	188.55(59.34–3793.51)	−2.664	<0.001

^a^ Concentration of the EO that kills 50% of the exposed flies. ^b^ Concentration of the EO that kills 95% of the exposed flies. Data are expressed as µL L^−1^ air. In bracket, there is a confidence interval. Pearson goodness of fit test: *χ2* = 56.151, df = 11, *p* < 0.001. Since the significance level (*p*) is less than 0.150, a heterogeneity factor is used in the calculation of confidence limits.

**Table 5:** Minimum inhibitory concentration (MIC) and minimum lethal concentration (MLC) values of the essential oils of *Allium sativum*, *Rosmarinus officinalis*, and *Salvia officinalis* against *Escherichia coli*, *Bacillus subtilis*, *Streptococcus aureus*, *Candida albicans,* and *Salmonella enterica* microbial strains.

Microorganism	*A. sativum*		*R. officinalis*		*S. officinalis*	
	MIC	MLC	MIC	MLC	MIC	MLC
*E. coli*	1.25 ^a^	1.25	2.50	2.50	5.00	5.00
*B. subtilis*	<0.63	<0.63	1.25	2.50	<0.63	<0.63
*S. aureus*	<0.63	<0.63	2.50	5.00	<0.63	<0.63
*C. albicans*	1.25	5.00	1.25	1.25	>10.00	>10.00
*P. aeruginosa*	1.25	1.25	2.50	2.50	<0.63	<0.63
*S. enterica*	10.00	10.00	>10.00	>10.00	>10.00	>10.00

^a^, Values are given as μL mL^−1.^

## Supplementary Materials

The following are available online at https://www.mdpi.com/2075-4450/11/3/143/s1. Figure S1: Disposition in the cage bottom of the embedding moulds filled by meshed meat treated with one of the tested essential oils (EOs) for the oviposition deterrence test. Each unit of four moulds treated with different EO concentrations (0.0, 1.2, 2.5, and 5 μL cm^−2^) was repeated at each of the cage corner.
