# Supplementary material for: Allium sativum, Rosmarinus officinalis, and Salvia officinalis Essential Oils: A Spiced Shield against Blowflies
Source: Insects. 2020 Feb 25;11(3):143. doi: 10.3390/insects11030143 (PMC7143423; doi:10.3390/insects11030143)
Supplement: Supplementary file 1 [file insects-11-00143-s001.pdf]

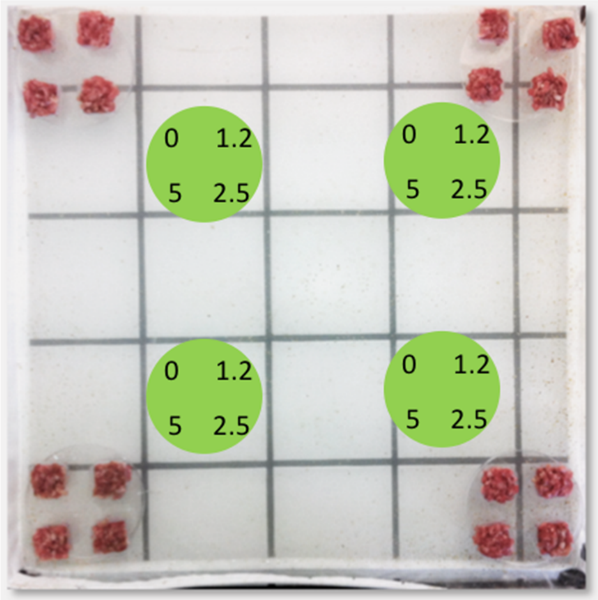

S1. Disposition in the cage bottom of the embedding moulds filled by meshed meat treated with one of the tested essential oils (EOs) for the oviposition deterrence test. Each unit of four moulds treated with different EO concentrations (0.0, 1.2, 2.5, and 5 µL cm^{-2}) was repeated at each of the cage corner.
